# Different classes of videoscopes and direct laryngoscopes for double-lumen tube intubation in thoracic surgery: A systematic review and network meta-analysis

**DOI:** 10.1371/journal.pone.0238060

**Published:** 2020-08-28

**Authors:** Young Sung Kim, Jihyun Song, Byung Gun Lim, Il Ok Lee, Young Ju Won

**Affiliations:** Department of Anesthesiology and Pain Medicine, Korea University Guro Hospital, Seoul, Republic of Korea; Zagazig University, EGYPT

## Abstract

**Background:**

Double-lumen tube is commonly used in thoracic surgeries that need one-lung ventilation, but its big size and stiff structure make it harder to perform intubation than a conventional tracheal intubation tube.

**Objectives:**

To investigate the effectiveness and safety of videoscopes for double-lumen tube insertion. The primary outcome was the success rate of first attempt intubation. Secondary outcomes were intubation time, malposition, oral mucosal damage, sore throat, and external manipulation.

**Design:**

Systematic review and network meta-analysis

**Data sources:**

Databases (Pubmed, Embase, Cochrane, Kmbase, Web of science, Scopus) up to June 23, 2020 were searched.

**Eligibility:**

Randomized controlled trials comparing different videoscopes for double-lumen tube intubation were included in this study.

**Methods:**

We classified and lumped the videoscope devices into the following groups: standard (non-channeled) videolaryngoscope, channeled videolaryngoscope, videostylet, and direct laryngoscope. After assessing the quality of evidence, we statistically analyzed and chose the best device based on the surface under the cumulative ranking curve (SUCRA) by using STATA software (version 16).

**Results:**

We included 23 studies (2012 patients). Based on the success rate of the first attempt, a rankogram suggested that the standard videolaryngoscope (76.4 of SUCRA) was the best choice, followed by videostylet (65.5), channeled videolaryngoscope (36.1), and direct laryngoscope (22.1), respectively. However, with regard to reducing the intubation time, the best choice was videostylet, followed by a direct laryngoscope, channeled videolaryngoscope, and standard videolaryngoscope, respectively. Direct laryngoscope showed the lowest incidence of malposition but required external manipulation the most. Channeled videolaryngoscope showed the highest incidence of oral mucosal damage, but showed the lower incidence of sore throat than standard videolaryngoscope or direct laryngoscope.

**Conclusion:**

Most videoscopes improved the success rate of double-lumen tube intubation; however, they were time-consuming (except videostylet) and had a higher malposition rate than the direct laryngoscope.

## Introduction

Double-lumen tube (DLT) is commonly used in thoracic surgeries that require one-lung ventilation. However, the intubation of DLT is challenging because it is much larger and stiffer in structure than a conventional single-lumen tracheal tube [1]. Moreover, patients undergoing thoracic surgery usually have limited tolerance for apnea; thus, precise and rapid tube insertion is a priority in such patients [2].

Over the last few decades, we have used direct laryngoscopes such as the Macintosh laryngoscope (Mc) in DLT intubation. Glidescope was introduced in the early 2000s [3], and since then, several video-assisted intubation devices have been introduced and have played an essential role in the airway management of patients with DLT. The American Society of Anesthesiologists (ASA) difficult airway algorithm recommended the use of video-assisted laryngoscopy as the initial approach to intubation in difficult airways [4]. However, the choice of an appropriate type of videoscope in a particular circumstance remains controversial.

Most videolaryngoscopes may be classified into a non-channeled or channeled type [5, 6]. Standard videolaryngoscopes (SVs), including the earlier versions of videolaryngoscopes such as Glidescope, were designed as non-channeled type. More recently, channeled videolaryngoscopes (CVs) have been developed. Compared to SV, CV has a unique feature. The right edge of the blade of CV has a longitudinal trough, which facilitates the approach of the tracheal tube tip toward the glottis.

The third type of videoscopes used in DLT intubation was a stylet videoscope (stylet V) [7, 8]. Unlike the other devices described above, stylet V is an intubating stylet with a similar structure as the lighted stylet (light wand) and similar features as the rigid videoscopes.

Although prior studies showed that videolaryngoscopes might provide an improvement in the intubation condition with a high success rate [9], there were many indicators for evaluating devices for DLT intubation, and it was challenging to identify the best device in the absence of a review study to distinguish these devices in terms of applicability in these special circumstances. We conducted network meta-analysis, in which multiple treatments are being compared, to assess the effectiveness, with regard to factors such as success rate of the first attempt of intubation, intubation time, and adverse event rates of intubating devices (Mc, SV, CV, and stylet V) as a strategy for selecting the right device for DLT intubation.

## Methods

Institutional Review Board approval did not apply to this systematic review (SR) and network meta-analysis (NMA), performed to compare the success rate of the first attempt; intubation time of the double-lumen endobronchial tube; and incidences of hoarseness, sore throat, malposition (contralateral side insertion), and external manipulation during intubation, among different videoscope devices used for direct laryngoscopy in patients who underwent surgeries under general anesthesia. This SR was registered on the PROSPERO (CRD42019124766; www.crd.york.ac.uk/PROSPERO) on April 3, 2019. The study protocol was based on the Cochrane Review Methods, and presented following the Preferred Reporting Items for Systematic Reviews and Meta-analyses (PRISMA) guidelines for reporting an NMA [10, 11]. We searched multiple comprehensive databases for literature regarding double-lumen endotracheal intubation using several types of videoscopes (including CEL-100, Glidescope, McGrath, Storz C-Mac, Airtraq DL, Pentax AWS-200, King Vision Video Laryngoscope, Optiscope, Shikani, and Trachway), and direct laryngoscopy (Macintosh laryngoscope; Mc group). The videoscope devices were classified and lumped into the following three groups based on their shapes and features: SV group for standard non-channeled blade videolaryngoscopes, CV group for channeled videolaryngoscopes, and stylet V group for videostylets.

### Database and literature sources

Until January 8, 2019, we searched for randomized controlled trials (RCTs) in PubMed, EMBASE, Cochrane Controlled Trials Register and Cochrane Database of Systematic Reviews, KMbase, Web of Science, and Scopus databases, that compared videoscopes or direct laryngoscopes for DLT intubation in patients undergoing general anesthesia. We searched for the following keywords in each database: general anesthesia, intubation, double-lumen, laryngoscopes, direct laryngoscope, video-laryngoscope, and thoracic surgery (S1 Appendix). After the initial electronic search, we evaluated the identified studies and performed a manual search using Google Scholar. To identify unpublished or ongoing studies, we searched the World Health Organization International Clinical Trials Registry Platform and the ClinicalTrials.gov website. The articles identified were assessed individually for inclusion in the analysis. We did not apply any language restriction to our search. We conducted a search again on the 23 June 2020. Subsequently, the information in the manuscript (‘Database and literature sources’ paragraph in the material and methods section and the 1st paragraph of result section), which was highlighted, and all results and figures were updated. Among the studies which were additionally found, 3 further studies were selected and included in the final analysis. Therefore, the new search affects the minor results but no effect on conclusions of our study.

### Study selection

A decision regarding the inclusion of studies in the analysis was made by two independent reviewers (YSK and YJW) based on the predefined inclusion criteria. Studies were selected after a two-level screening. First, we screened the titles and abstracts of the identified studies. Second, we reviewed the full texts. Discrepancies between the reviewers were resolved through discussions. Studies that met the following criteria were included in our NMA: (1) RCTs performed in patients who underwent thoracic surgeries under general anesthesia; (2) studies comparing types of videoscope devices and direct laryngoscopy during double-lumen endobronchial tube insertion; and (3) studies involving the assessment of the success rate of the first attempt, intubation time of double-lumen endobronchial tube, and incidences of tube malposition, oral mucosal damage, sore throat, and external manipulation during intubation.

### Data extraction

The two reviewers independently extracted data from each study by using a predefined data extraction form. Any disagreement unresolved by the discussion was resolved in consultation with a third reviewer (BGL).

The following variables were extracted from the studies: (1) mean and standard deviation of reduction in intubation time of double-lumen endobronchial tube as continuous variables, and dichotomous variables including the success rate of the first attempt, incidence of tube malposition (intubated to the contralateral side), oral mucosal damage, sore throat, and external manipulation during intubation; (2) demographic and clinical characteristics, such as age, sex, and number of patients in the different intubation device groups; (3) first author, country, and year of publication; and (4) method of assessment. If the above variables were not found in the articles, we requested the data from their authors via email.

### Assessment of methodological quality

The two reviewers independently assessed the methodological quality of each study by using the Cochrane Collaboration’s tool for assessing the risk of bias (Review Manager Version 5.3, The Cochrane Collaboration, Oxford, UK). This tool is widely used to assess the methodological quality of RCTs and consists of the following six items: random sequence generation, allocation concealment, blinding of participants and personnel, blinding of outcome assessment, incomplete outcome data, and selective outcome reporting. The risk of bias was classified as high, low, or unclear [10]. Any disagreements between the reviewers were resolved through discussions or by the third reviewer.

### Statistical analysis

We used STATA software (version 16; StataCorp LP, College Station, Texas, USA) and NMA graphical tools by Chaimani et al. [12] for the multiple-treatment comparison NMA. It was a generalized meta-analysis method that included both direct RCT comparisons and indirect comparisons of treatments.

The primary outcome of this SR was the first attempt success rate of double-lumen endobronchial tube insertion among the several types of videoscope devices and direct laryngoscopy. Secondary outcomes were intubation time of double-lumen endobronchial tube and rates of adverse events such as incidences of tube malposition, oral mucosal damage, sore throat, and external manipulation.

For contribution assessment, we derived the direct estimates using a comparison-specific random-effects model. The plausibility of the transitivity assumption was assessed based on the design characteristics and the methodology of the studies included in the NMA, as recommended [13]. We evaluated the consistency assumption for the entire network using a design-by-treatment interaction model and examined each closed loop in the network to evaluate local inconsistencies between the direct and indirect effect estimates for the same comparison. The quadratic loop was not included in our network. In each loop, we evaluated the inconsistency factor (IF) as the absolute difference (95% confidence interval [CI]) and using z-test between the direct and indirect estimates for each paired comparison in the loop. The IF is the logarithm of the ratio of two odds ratios (RoRs) from direct and indirect evidence in the loop; RoRs close to 1 indicate that the two sources are in agreement [14]. The consistencies between the direct and indirect comparisons for all closed loops were evaluated [15]. Testings for inconsistency were evaluated as χ^2^ using a global approach. The estimated pair-wise summary effects of each outcome were evaluated using 95% CIs and predictive intervals (PrI) of the estimates. The mean summary of effects was presented together with the PrI data to facilitate the interpretation of results considering the magnitude of heterogeneity between studies.

We used the surface under the cumulative ranking curve (SUCRA) values calculated from the rankograms to present the hierarchy of interventions for the success rate at the first attempt, intubation time, and the rates of adverse events and external manipulation. A rankogram represented the probabilities for treatments to assume any possible ranks. SUCRA was the relative ranking measure that accounted for the uncertainty in the treatment order, which in turn accounted for both the location and variance of all relative treatment effects. A SUCRA value of 1 (or 100%) meant that an intervention was certain to be the best (i.e., always ranked first), whereas 0 (or 0%) meant the worst. A higher SUCRA value was regarded as a better result for individual interventions [16].

## Results

### Identification of studies

**Fig 1:**
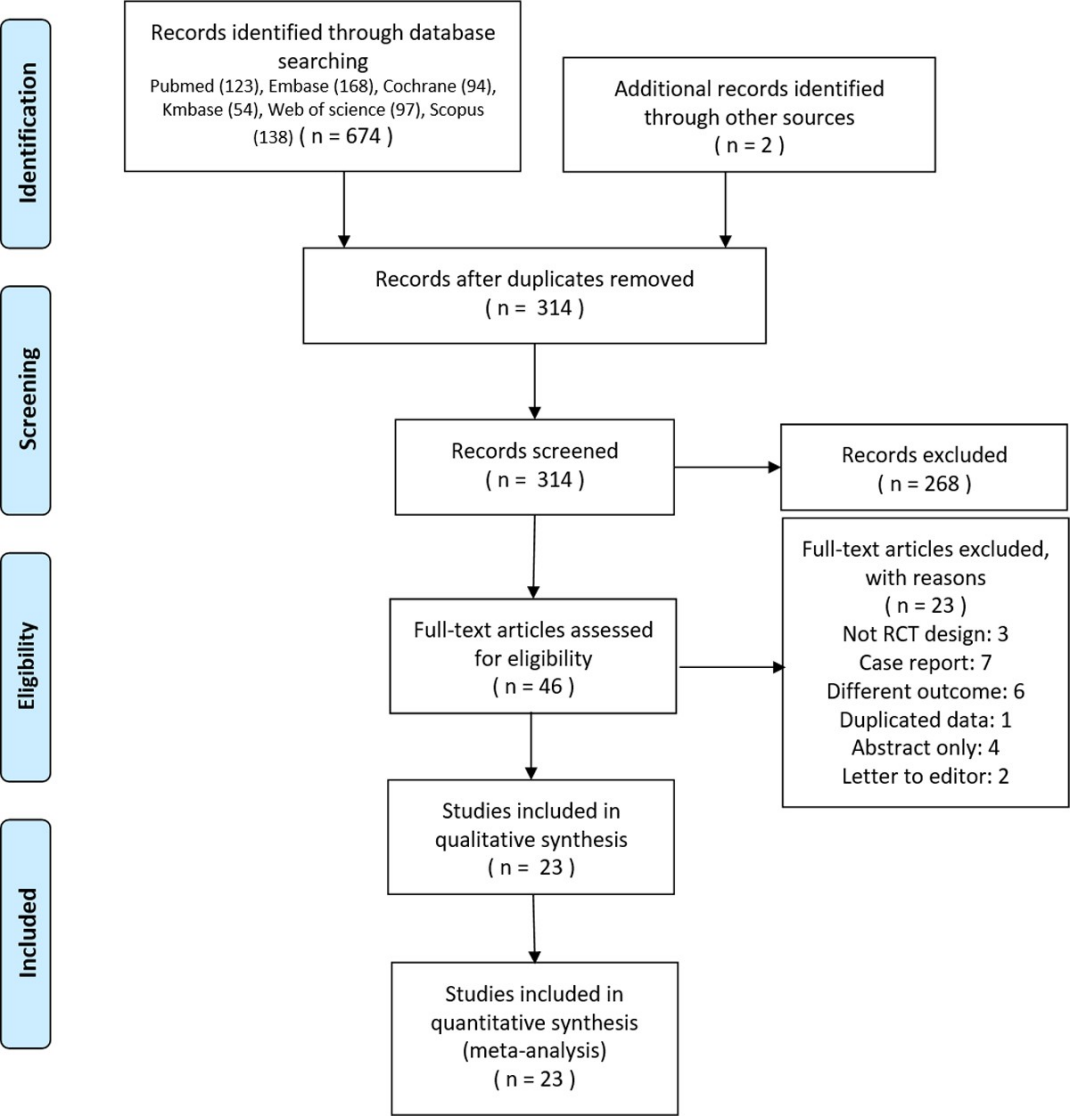
PRISMA flow diagram. A total of 23 randomized controlled trials of double-lumen tube intubation with videoscopes were included in this network meta-analysis.

The search strategy details are provided in S1 Table. Searches of the databases yielded 676 articles. Of these, 630 publications were excluded, as it was clear from the title and abstract that they did not fulfill the selection criteria. For the remaining 46 articles, we obtained and scrutinized the full manuscripts to identify potentially relevant articles. Twenty-three articles were excluded as follows: three were not RCT designed studies, four were only published abstracts, two were letters to the editor, seven were case reports, six evaluated different outcomes to this study (for the transitivity assumption not to be violated), and one was a redundant publication. Therefore, the total number of studies included in the SR was 23 (Fig 1).

### Study characteristics

The 23 studies (total 2012 patients) were RCTs. The characteristics of these studies are summarized in Table 1 and S2 Table. One RCT was written in French [17], three RCTs were written in Chinese [18–20], and the other 19 studies were written in English.

**Table 1:** Study characteristics.

Study	Year	Origin	Publication language	Participants	Group	Level of operator experience
Ajimi et al.	2018	Japan	English	ASA 1–3, age 20–84 years	Airtraq n = 30	One senior anesthetist with at least > 200 DLT with Macintosh
					AWS-200 n = 30	
Bakshi et al.	2019	India	English	ASA 1–2, adults	McGrath MAC n = 37	Eleven nonexperts
					Macintosh n = 37	
Belze et al.	2017	France	English	not specified	Glidescope n = 36	Three anesthetists with at least 10 DLT with each device
					Airtraq n = 36	
Bensghir et al.	2010	Morocco	French	ASA 1–2, age > 18 years	Glidescope n = 34	Experienced
					Macintosh n = 34	
H. Kido et al.	2015	Japan	English	ASA 1–3, age 20–85 years	McGrath MAC n = 25	Anesthesia residents
					Macintosh n = 25	
Hamp et al.	2015	Austria	English	ASA 1–2, adults	Airtraq n = 17	Two experienced anesthetists
					Macintosh n = 20	
Hsu et al.	2012	Taiwan	English	ASA 1–2, adults	Glidescope n = 30	Two experienced anesthetists
					Macintosh n = 30	
Hsu et al.	2013	Taiwan	English	ASA 1–3, age > 18 years	Trachway n = 30	Two experienced anesthetists
					Macintosh n = 30	
Huang et al.	2020	China	English	ASA 1-2.age 18–75 years	Glidescope n = 30	Five anesthesiologists with 10 years’ working experience
					C-MAC n = 30	
					Macintosh n = 30	
Jiang et al.	2011	China	Chinese	ASA 1–2, age > 18 years	Airtraq n = 29	Not mentioned
					Macintosh n = 29	
Lin et al.	2012	China	English	ASA 1–3, adults	CEL-100 n = 83	Three experienced anesthetists
					Macintosh n = 82	
M.R. El-tahan et al.	2018	Saudi Arabia	English	ASA 2–3, age 18–70 years	Macintosh n = 32	Anesthesia consultants, specialists, and trainees
					Glidescope n = 34	
					Airtraq n = 35	
					King Vision n = 32	
Risse et al.	2020	Germany	English	ASA 1–4, adults	Glidescope n = 35	Three experienced physicians
					Macintosh n = 35	
Russell et al.	2013	Canada	English	ASA 2–4, age > 18 years	Glidescope n = 35	30 novice anesthetists
					Macintosh n = 35	
Shah et al.	2016	India	English	ASA 1–3, age 18–80 years	Storz C-Mac D-blade n = 29	Two experienced anesthetists
					Macintosh n = 30	
Wan et al.	2016	China	English	ASA 1–3, age 18–70 years	McGrath n = 45	Not mentioned
					Airtraq n = 45	
Wasem et al.	2013	Germany	English	ASA 1–2, Age 18–78 years	Airtraq n = 30	Two experienced anesthetists
					Macintosh n = 30	
Xu et al.	2015	China	Chinese	ASA 1–3, age 18–70 years	Shikani n = 30	An experienced anesthetist (at least 300 times)
					Macintosh n = 30	
Yang et al.	2013	Republic of Korea	English	ASA 1–3, age 18–80 years	OptiScope n = 198	Five anesthetists with more experience
					Macintosh n = 199	
Yao et al.	2015	China	English	ASA 1–3, age 18–70 years	McGrath series 5 n = 48	Three senior anesthetists with extensive experience
					Macintosh n = 48	
Yi et al.	2013	China	Chinese	ASA 1–3, age 18–75 years	Macintosh n = 35	Not mentioned
					Glidescope n = 35	
Yi et al.	2015	China	English	ASA 1–2, age 18–75 years	Airtraq n = 36	One experienced anesthetist
					Glidescope n = 35	
Yoo et al.	2018	Republic of Korea	English	ASA 1–2, age 19–60 years	Macintosh n = 22	One experienced anesthetist
					McGrath n = 22	

ASA numbers refer to the American Society of Anesthesiologists physical status classification.

In the 23 RCTs, we identified a total of 11 different intubating devices, that were further classified and lumped into four groups based on their shapes and features: videolaryngoscope with guiding channel (channeled videolaryngoscope; CV), non-channeled videolaryngoscope (standard videolaryngoscope; SV), videostylet (stylet V), and Macintosh direct laryngoscope (Mc) [21–23]. We lumped Airtraq, AW, and KVL in the CV group; CEL-100, Glidescope, McGrath, and C-MAC in the SV group; and OptiScope, Shikani, and Trachway in the stylet V group.

#### Risk of bias assessment see Fig 2

**Fig 2:**
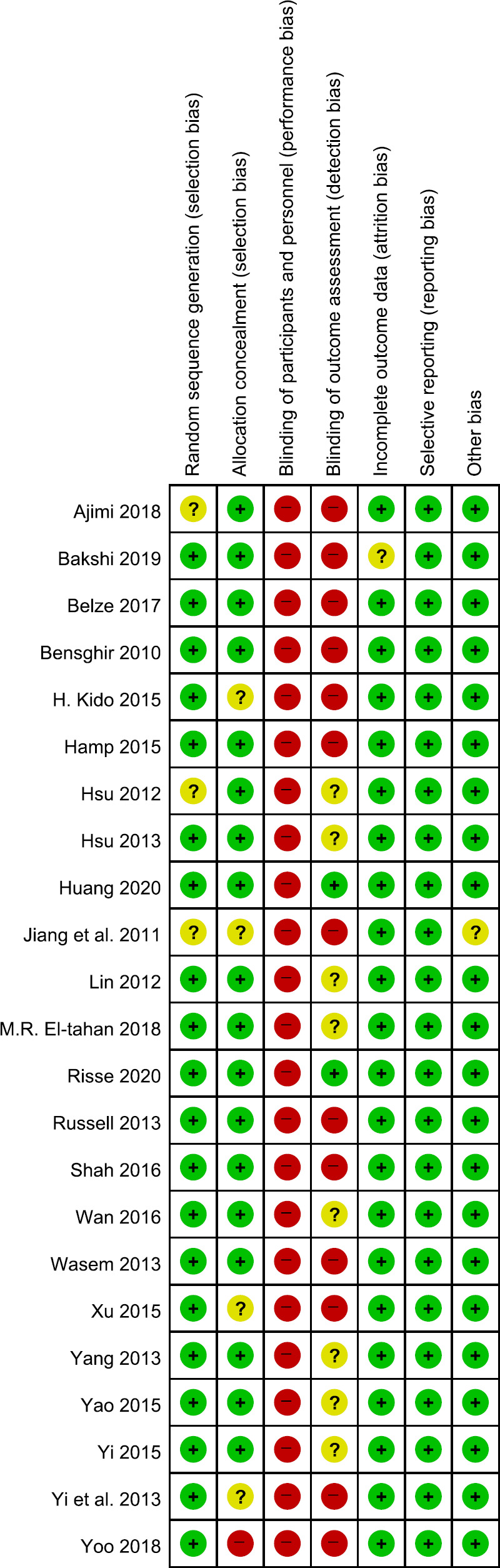
Risk of bias assessments.

For randomization, all of the 23 studies in our SR reported that the study was randomized; 20 of them (87%) reported that the method of random sequence generation was applied, whereas the other three studies (13%) did not provide adequate information [19, 24, 25]. The allocation concealment was adequately reported in 18 studies, four studies reported it inadequately [1, 18–20], and one study checked as high risk [26]. For blinding, given that different devices were used in almost all RCT studies, all studies (100%) were evaluated as high risk in blinding of participants and personnel to the intervention of the studies (performance bias). Eight studies (35%) that reported blinding of the outcome assessors were assessed as having unclear risk of bias [7, 8, 25, 27–31], and the other thirteen studies (57%) were assessed as having high risk of bias [1, 17–20, 24, 26, 32–37]. For incomplete outcome data report, Twenty-two studies (96%) reported the completeness of outcome data for each main outcome and were assessed as having a low risk of bias. One study (4%) checked as unclear risk [32]. All studies (100%) were assessed as having a low risk of bias about Selective reporting and Other potential sources of bias.

### Synthesis of results

Before conducting the NMA, we evaluated the transitivity assumption by examining the comparability of the risk of bias as a potential treatment-effect modifier across comparisons. After confirming that the transitivity assumption was not violated, we conducted the NMA and consistency assessments.

**Fig 3:**
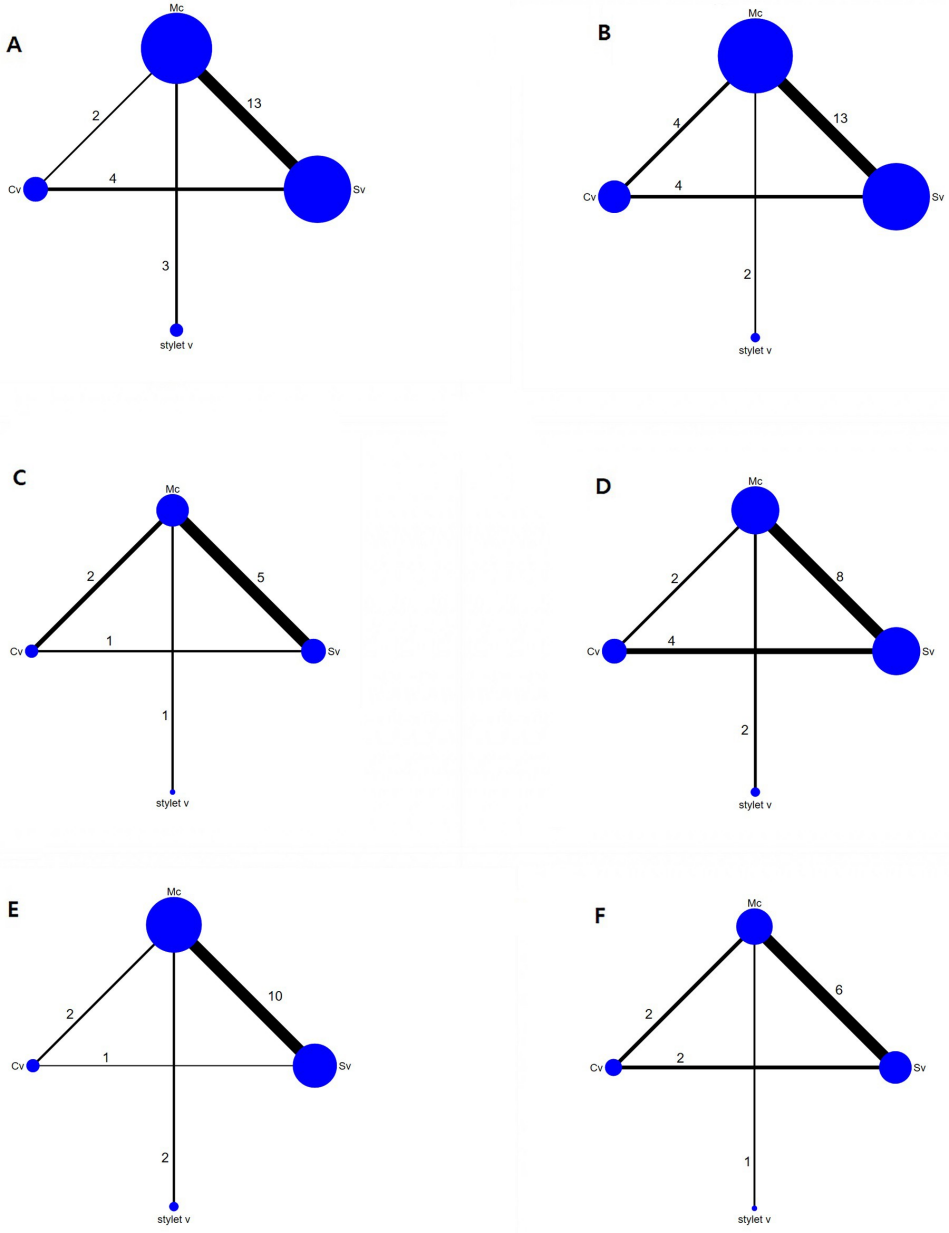
Evidence network plots for the outcomes showing the four types of laryngoscopes included in the network meta-analysis. The size of the nodes corresponds to the total number of studies with each device. The thickness of the lines is proportional to the number of studies making this comparison. (A) The success rate of the first attempt, (B) intubation time, (C) malposition, (D) sore throat, (E) oral mucosal damage, and (F) external manipulation. Mc, Macintosh (direct) laryngoscope; SV, standard non-channeled videolaryngoscope; CV, channeled videolaryngoscope; stylet V, videostylet.

**Fig 4:**
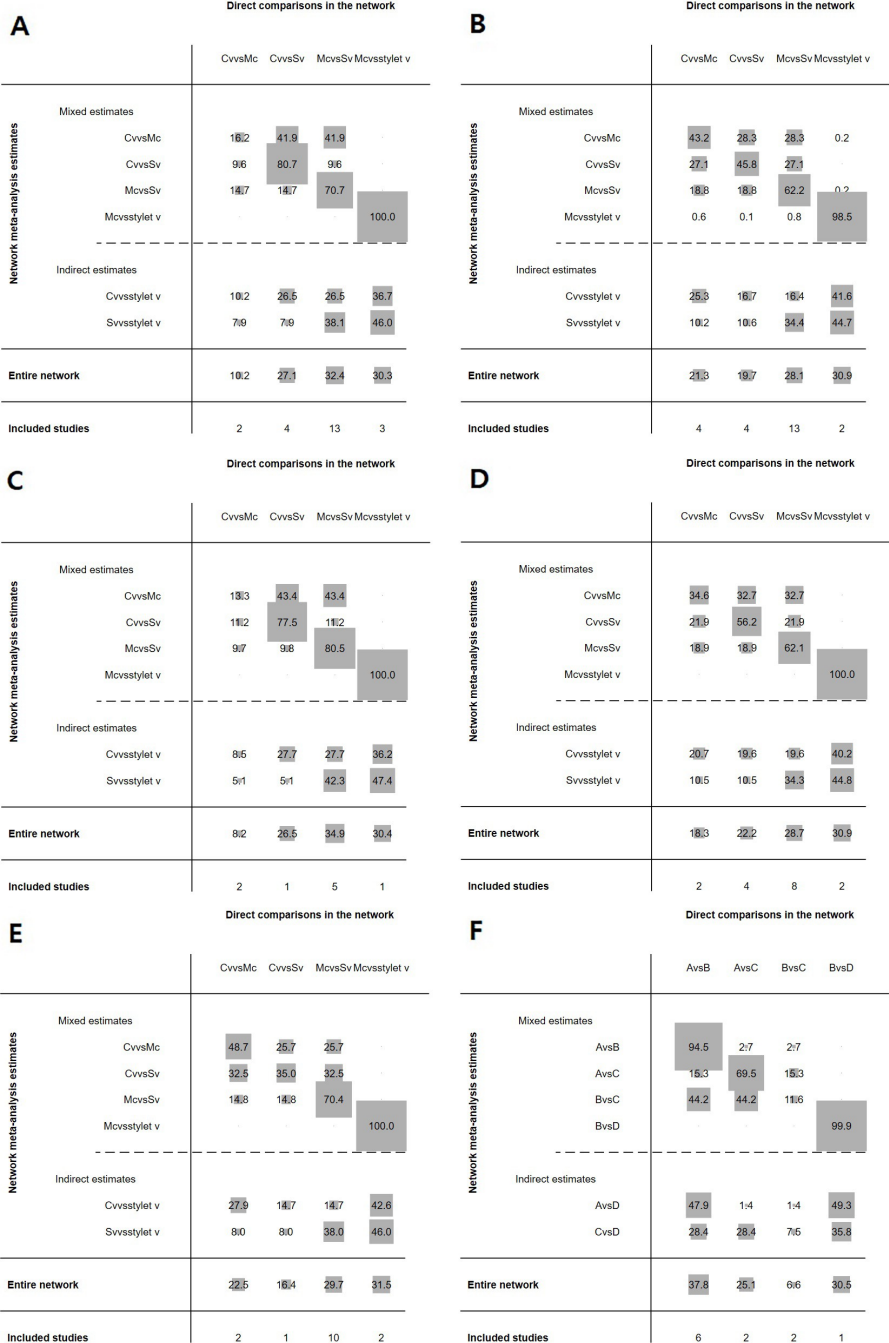
Contribution plot for each direct comparison. The rows correspond to the mixed and indirect evidence, whereas the columns correspond to the direct evidence. The percentage contribution of each direct comparison to the network summary is presented in the entire network row. The sizes of the boxes are proportional to the percentage contribution of each direct estimate to the network meta-analysis estimates and to the entire network. The last row shows the number of direct comparisons included. (A) The success rate of the first attempt, (B) intubation time, (C) malposition, (D) sore throat, (E) oral mucosal damage, and (F) external manipulation. Mc, Macintosh (direct) laryngoscope; SV, standard non-channeled videolaryngoscope; CV, channeled videolaryngoscope; stylet V, videostylet.

**Fig 5:**
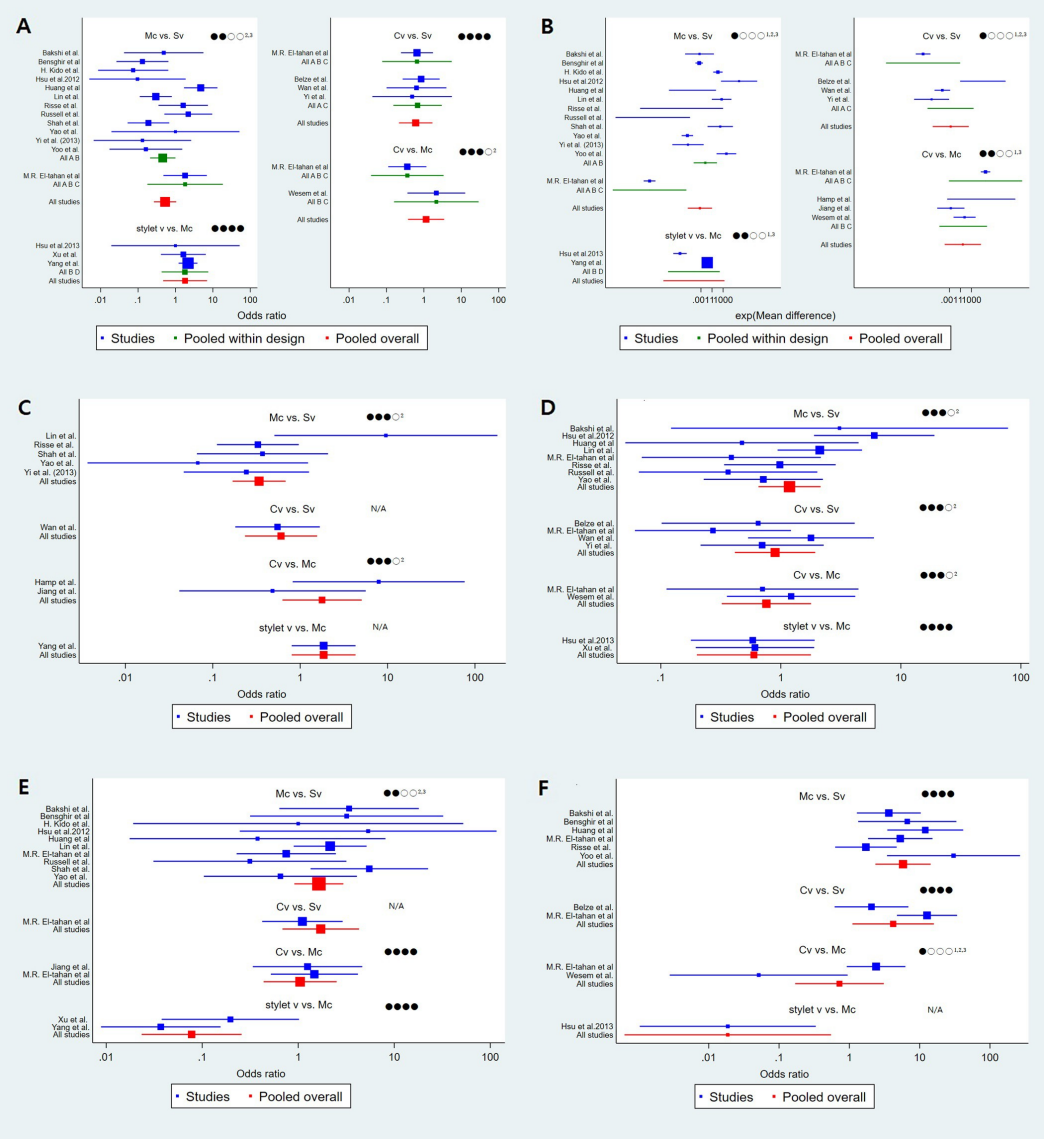
The estimated pair-wise summary effects of outcomes that show the 95% CI and PrI of the estimates and the GRADE score. A GRADE score was assessed in each comparison. ^1^high inconsistency, ^2^high indirectness, ^3^high imprecision (wide CI). (A) The success rate of the first attempt, (B) intubation time, (C) malposition, (D) sore throat, (E) oral mucosal damage, and (F) external manipulation. Mc, Macintosh (direct) laryngoscope; SV, standard non-channeled videolaryngoscope; CV, channeled videolaryngoscope; stylet V, videostylet.

**Fig 6:**
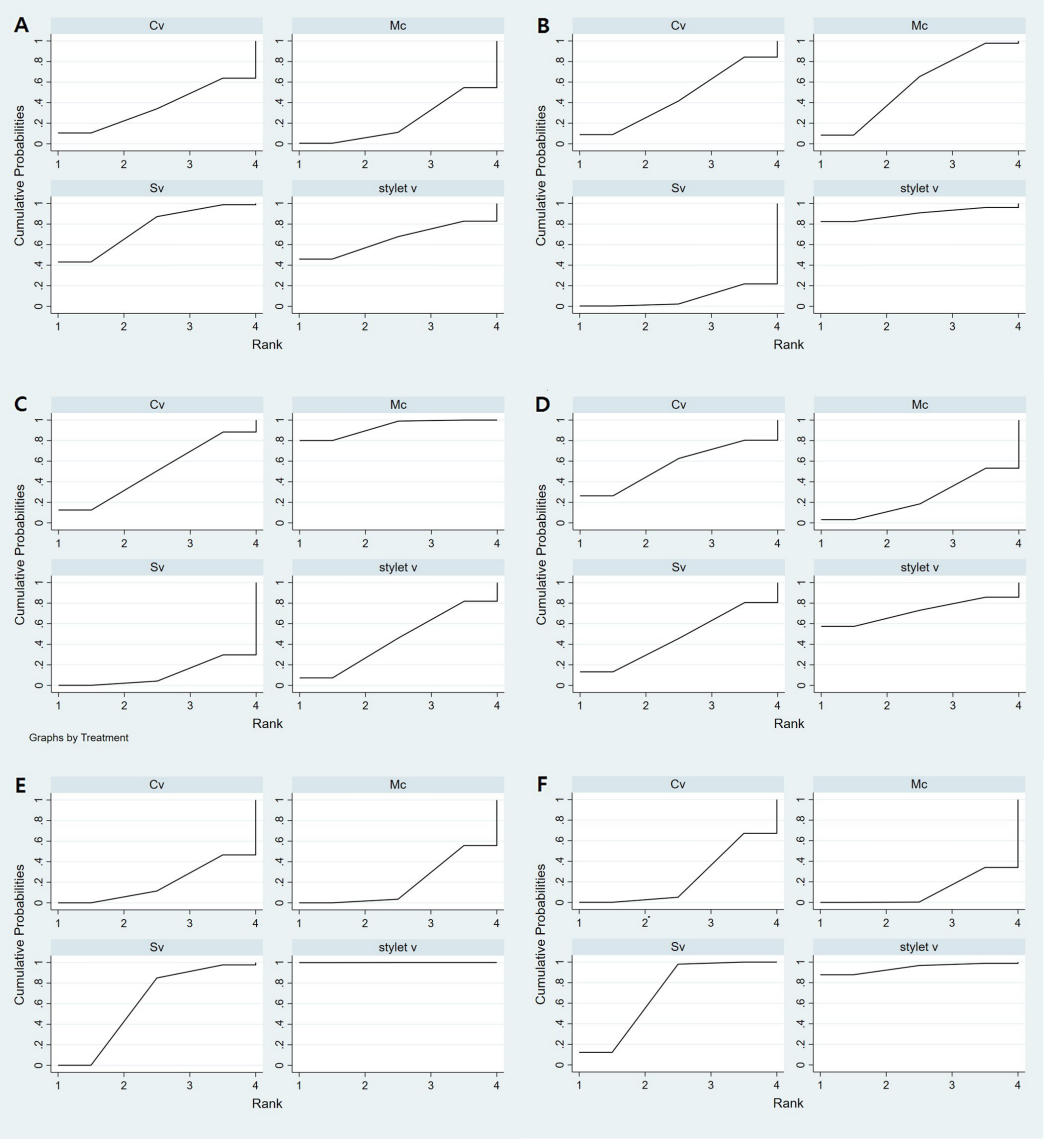
The cumulative ranking curve of the outcomes of the different laryngoscopes. The surface under the cumulative ranking curve (SUCRA) represents the ranking of devices. A higher SUCRA suggests a higher probability of being a good device. (A) The success rate of the first attempt, (B) intubation time, (C) malposition, (D) sore throat, (E) oral mucosal damage, and (F) external manipulation. Mc, Macintosh (direct) laryngoscope; SV, standard non-channeled videolaryngoscope; CV, channeled videolaryngoscope; stylet V, videostylet.

For the outcomes of each pooled data, we presented the network geometry (Fig 3), the direct to indirect league table (Fig 4), the estimated pair-wise summary effects of outcomes that showed the 95% CI and PrI of the estimates and GRADE analysis (Fig 5), and SUCRA ranking (Fig 6).

### Primary endpoint

#### Success rate of the first attempt intubation of DLT

Four groups of devices from 21 studies were available for the analysis [1, 7, 8, 17, 18, 20, 24–33, 35–39]. Eligible comparisons in the NMA are shown in Figs 3A and 4A. All the 95% CIs for RoR were compatible with the zero inconsistency (RoR = 1). The evaluation of the network inconsistency using the design–by-treatment interaction model suggested no evidence of a statistically significant inconsistency (χ^2^(3) = 1.73, p = 0.63), so we analyzed using the consistency model. The quality of evidence assessed by GRADE analysis varied from moderate to very high for the NMA estimates (Fig 5A). A rankogram suggested that the SV (76.4 of SUCRA) was the best choice, followed by stylet V (65.5), CV (36.1), and Mc (22.1), respectively (Fig 6A).

### Secondary endpoints

#### Intubation time

Four groups of devices from 22 studies were available for the analysis [1, 7, 8, 17, 19, 20, 25–39]. Eligible comparisons in the NMA are shown in Figs 3B and 4B. The evaluation of network inconsistency by using the design–by treatment interaction model suggested no evidence of a statistically significant inconsistency (χ2(3) = 8.03, p = 0.045); therefore, we analyzed using the inconsistency model. The quality of evidence varied from low to moderate for NMA estimates (Fig 5B). The best choice was stylet V (90.3), followed by Mc (57.6), CV (45.5), and SV (6.6) (Fig 6B and S1 Fig).

#### Malposition

Four groups of devices from 9 studies were available for the analysis [8, 19, 20, 28–30, 34, 36, 39]. Eligible comparisons in the NMA are shown in Figs 3C and 4C. The evaluation of network inconsistency using the design–by-treatment interaction model suggested no evidence of a statistically significant inconsistency (χ2(1) = 0.10, p = 0.757); therefore, we analyzed using the consistency model. The quality of evidence was high for the NMA estimates (Fig 5C). DLT malposition occurred the least in Mc (93.2) compared to the videoscopes (50.5 for CV, 44.0 for stylet V, and 12.3 for SV) (Fig 6C).

#### Sore throat

Four groups of devices from 14 studies were available for the analysis [7, 18, 20, 25, 27–30, 32, 33, 35, 37–39]. Eligible comparisons in the NMA are shown in Figs 3D and 4D. The evaluation of network inconsistency using the design–by-treatment interaction model suggested no evidence of a statistically significant inconsistency (χ2(3) = 2.70, p = 0.441); therefore, we analyzed using the consistency model. The quality of evidence varied from high to very high for the NMA estimates (Fig 5D). Stylet V was the best choice for sore throat prevention; sore throat incidence using CV was lower than that using SV and Mc (72.1 for stylet V, 56.6 for CV, 46.5 for SV, and 24.9 for Mc) (Fig 6D).

#### Oral mucosal damage

Four groups of devices from 13 studies were available for the analysis [1, 8, 17–19, 25, 27–29, 32, 35, 36, 38]. Eligible comparisons in the NMA are shown in Figs 3E and 4E. The evaluation of network inconsistency using the design–by-treatment interaction model suggested no evidence of a statistically significant inconsistency (χ2(2) = 1.58, p = 0.454); therefore, we analyzed using the consistency model. The quality of evidence varied from moderate to very high for the NMA estimates (Fig 5E). Stylet V was the best choice for the prevention of oral mucosal damage; oral mucosal damage showed most frequently in CV among the devices (99.9 for stylet V, 60.8 for SV, 19.9 for Mc, and 19.4 for CV) (Fig 6E).

#### External manipulation

Four groups of devices from 9 studies were available for the analysis [7, 17, 26, 27, 32, 33, 37–39]. Eligible comparisons in the NMA are shown in Figs 3F and 4F. The evaluation of network inconsistency using the design–by-treatment interaction model suggested no evidence of a statistically significant inconsistency (χ2(3) = 5.25, p = 0.154), so we analyzed using the consistency model. The quality of evidence varied from low to very high for the NMA estimates (Fig 5F). Stylet V (94.4) was the best choice and required the least amount of external manipulation, followed by SV (70.1), CV (24.1), and Mc (11.4), respectively (Fig 6F).

## Discussion

In this review, we showed that SV was the most useful device in terms of the success rate of the first attempt at DLT intubation. Not only SV but also the other videoscopes showed better success rates than Mc. The results were consistent with the previous studies on DLT intubation [1, 8, 17, 18, 20, 25, 26, 29, 30, 32, 36, 37]. However, the best choices differed according to the indicators. Videolaryngoscopes, especially SV, seemed time-consuming compared to Mc. All videoscopes showed a higher probability in tube malposition risk than Mc. The oral mucosal damage occurred most frequently with the CV, while sore throat and external manipulation occurred most frequently in Mc.

During the insertion of the endotracheal tube using the SV or CV, there is a blind period when the tip of the endotracheal tube does not appear on the video screen. During this period, the endotracheal tube is moved with an exploratory feature, which may cost time and cause endotracheal tube related injuries [6]. The main disadvantage of non-channel blades is that it is more difficult to handle tracheal tube and videolaryngoscope at the same time while maintaining the best glossiness view on the screen. In this scenario, a guidance ‘channel’ may be helpful in handling the endotracheal tube. This assumption was consistent with our results of higher SUCRAs in intubation time and sore throat in the CV than in the SV. Biro and Martin [6] reported that the channeled videolaryngoscopy required more time in glottis recognition, but lesser total intubation time than the non-channeled videolaryngoscopy.

Moreover, a two-dimensional (2D) view on a flat-screen may cause confusion in the depth perception than the direct view [40, 41]. Fazlyyyakhmatov et al. [42] assessed cortical activity in the process of depth perception of 2D images. In stereoscopic vision, a few centimeters of the distance between the two eyes causes each eye to have a slightly different view of the same scene. The brain combines the two views into a single 3D image, which is called stereopsis. However, all images on a 2D view are located at the same distance from the observation point, which makes it challenging to structure the stereoscopic spatial perception of the images. Our result showed a higher probability of tube malposition in most videoscopes than the direct laryngoscope. Although there is insufficient evidence, it seems that the limitations of the 2D-view may have contributed to the tube malposition. In the fields of laparoscopic surgery, a three-dimensional (3D) display is used to improve the technical precision and hand-eye coordination [41]; however, no commercial product is available for 3D videolaryngoscopy. Anyway, higher malposition rate did not result in higher failure rate in the videoscopes compared to the direct laryngoscope. It was because the main etiology of failure was insertion failure to the endotracheal space rather than malposition.

Unlike other complications, oral mucosal damage occurred most frequently in CV. Oral mucosal damage seemed more likely to be caused by the laryngoscope blade rather than an endotracheal tube related injury possibly due to the large blade size as a result of the presence of the ‘channel.’

Interestingly, stylet V showed reliable results in overall indicators. The intubation time was the shortest among the devices. A possible explanation is that stylet V did not require the process of blade insertion during the DLT intubation. In addition, stylet V showed fewer complications (oral mucosal damage and sore throat) and lesser need for external manipulation than the other devices. These findings are very encouraging for stylet V; however, no study has compared it directly with the other videolaryngoscopes. Our results were calculated only through indirect comparisons in the open-loop. Further studies are required to compare and confirm the consistency of these results.

During this NMA, we carefully thought out the process of comparing several types of videoscope devices. Several systematic reviews and meta-analyses have led to some conflicting results regarding the comparative performance of these devices in specific situations. Firstly, adequate standards in various endpoints and definitions of difficult laryngoscopy and intubation were lacking. The word ‘difficulty’ is ambiguous and hard to define considering many factors, including previous history, anatomical changes, mouth opening, and failure despite a good view. The Cormack and Lehane system was known to be less relevant in videolaryngoscopy than in direct laryngoscopy [43]. Each instrument had its own characteristics in terms of blade size, blade angle, display, camera resolution, view angle, light type, weight, and use of a stylet. In the subgroup analysis within the SVs, we found a slightly lower SUCRA in the hyperangulated SV (glidescopes) than the other SVs; however, the difference was not prominent (data not shown). Furthermore, the patient groups (difficulty is expected or not expected) and the proficiency of the practitioners varied in the prior studies. Therefore, our analysis was inevitably heterogeneous, and our results for the performance may not be generalizable.

Despite these limitations, our study has many advantages. To the best of our knowledge, this NMA was the first attempt to analyze various indicators in assessing the DLT intubation comprehensively. Our results were informative in various clinical situations. In each case, it was important to set the right priorities for the success of the intubation, the time it took, and the possible complications. Although there are still limitations, the development and application of the videoscopes have significantly improved the options in difficult airway management. We suggest that preparing and becoming familiar with different types of videoscopes will be helpful in various clinical situations as well as during the double-lumen intubation. We expect that the development of 3D videoscopes may improve upon the shortcomings of the current videoscopes.

In conclusion, this NMA revealed that a standard blade videolaryngoscope was the best choice in terms of the success rate of the first attempt of DLT intubation, although it seemed time-consuming and had a higher malposition rate than the other devices. Most videoscopes improved the success rate of the DLT intubation but did not seem to reduce the incidence of tube malposition compared to the direct laryngoscope. Further RCTs are needed to compare the videostylet and videolaryngoscopes directly.

## Supporting information

S1 Fig Rankogram with inconsistency model for intubation time (See Fig 6B). (TIF)

S1 Table The search strategy details. (DOCX)

S2 Table Clinical characteristics for enrolled studies. (DOCX)

S1 File PRISMA NMA checklist. (DOCX)

S1 Appendix (DOCX)
