# Supplementary figures and images for: Different classes of videoscopes and direct laryngoscopes for double-lumen tube intubation in thoracic surgery: A systematic review and network meta-analysis
Source: PLoS One. 2020 Aug 28;15(8):e0238060. doi: 10.1371/journal.pone.0238060 (PMC7455027; doi:10.1371/journal.pone.0238060)

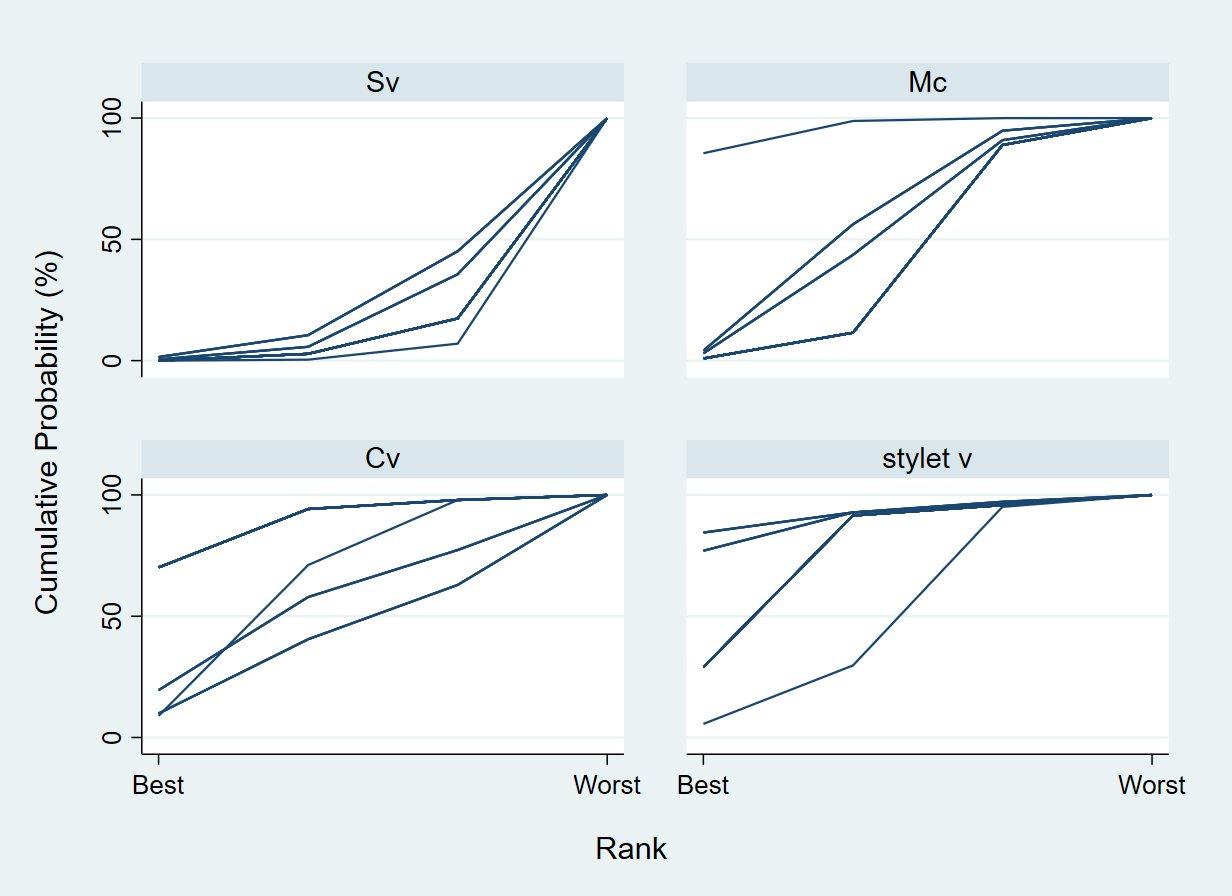

Supplement: S1 Fig — (TIF) [file pone.0238060.s001.tif]
