# Supplementary material for: Different classes of videoscopes and direct laryngoscopes for double-lumen tube intubation in thoracic surgery: A systematic review and network meta-analysis
Source: PLoS One. 2020 Aug 28;15(8):e0238060. doi: 10.1371/journal.pone.0238060 (PMC7455027; doi:10.1371/journal.pone.0238060)
Supplement: S1 Table — (DOCX) [file pone.0238060.s002.docx]

S1 Table. The search strategy details

| PICO | PubMed | Embase | Cochrane | WoS |
| --- | --- | --- | --- | --- |
| Population/ Problems | (Intubation, Intratracheal[MH] OR Intubat*[tw]) AND (double lumen[tw] OR dual lumen[tw]) | ('endotracheal intubation'/exp OR 'Nasotracheal Intubation'/exp OR intubat*:ti,ab) AND ('double lumen tube'/exp OR (double NEAR/3 lumen OR dual NEAR/3 lumen):ti,ab) | ([mh "Intubation, Intratracheal"] OR Intubat*:ti,ab,kw) AND (double NEAR/3 lumen OR dual NEAR/3 lumen):ti,ab,kw | TS=(Intubat* AND (double NEAR/3 lumen OR dual NEAR/3 lumen)) |
| Intervention (MeSH/Emtree) | (Laryngoscopy[mh] OR Laryngoscopes[mh]) AND Video Recording[mh] | videolaryngoscopy/exp OR (laryngoscopy/exp AND videorecording/exp) | ([mh Laryngoscopy] OR [mh Laryngoscopes]) AND [mh "Video Recording"] |  |
| Intervention | videolaryngoscop*[tw] OR video-laryngoscop*[tw] OR VL[tw] OR VLS[tw] OR (video[tw] AND laryngoscop*[tw]) OR Airtraq[tw] OR Bullard[tw] OR Pentax[tw] OR Glidescope[tw] OR McGrath[tw] OR Storz[tw] OR Venner[tw] OR King Vision[tw] OR KingVision[tw] OR VividTrac[tw] OR CoPilot[tw] OR Ue scope[tw] OR stylet[tw] OR TruView[tw] OR Airway Scope[tw] OR AWS[tw] OR CEL[tw] OR C-MAC[tw] OR A.P. Advance[tw] OR EndoSTROB[tw] OR Glidescope[tw] OR VLP-100[tw] OR LMA CTrach[tw] | (videolaryngoscop* OR video-laryngoscop* OR VL OR VLS OR ((video OR indirect) NEAR/3 laryngoscop*) OR Airtraq OR Bullard OR Pentax OR Glidescope OR McGrath OR Storz OR Venner OR 'King Vision' OR KingVision OR VividTrac OR CoPilot OR 'Ue scope' OR stylet OR TruView OR 'Airway Scope' OR AWS OR CEL OR C-MAC OR 'A.P. Advance' OR EndoSTROB OR Glidescope OR VLP-100 OR 'LMA CTrach'):ti,ab | (videolaryngoscop* OR video-laryngoscop* OR VL OR VLS OR ((video OR indirect) NEAR/3 laryngoscop*) OR Airtraq OR Bullard OR Pentax OR Glidescope OR McGrath OR Storz OR Venner OR "King Vision" OR KingVision OR VividTrac OR CoPilot OR "Ue scope" OR stylet OR TruView OR "Airway Scope" OR AWS OR CEL OR C-MAC OR "A.P. Advance" OR EndoSTROB OR Glidescope OR VLP-100 OR "LMA CTrach"):ti,ab,kw | TS=(videolaryngoscop* OR video-laryngoscop* OR VL OR VLS OR ((video OR indirect) NEAR/3 laryngoscop*) OR Airtraq OR Bullard OR Pentax OR Glidescope OR McGrath OR Storz OR Venner OR "King Vision" OR KingVision OR VividTrac OR CoPilot OR "Ue scope" OR stylet OR TruView OR "Airway Scope" OR AWS OR CEL OR C-MAC OR "A.P. Advance" OR EndoSTROB OR Glidescope OR VLP-100 OR "LMA CTrach") |
| Comparison | direct laryngoscop*[tw] OR DL[tw] OR conventional laryngoscop*[tw] OR (Macintosh[tw] AND laryngoscop*[tw]) OR (Miller[tw] AND laryngoscop*[tw]) | (direct NEAR/3 laryngoscop* OR DL OR conventional NEAR/3 laryngoscop* OR Macintosh NEAR/3 laryngoscop* OR Miller NEAR/3 laryngoscop*):ti,ab OR 'device comparison'/exp | (direct NEAR/3 laryngoscop* OR DL OR conventional NEAR/3 laryngoscop* OR Macintosh NEAR/3 laryngoscop* OR Miller NEAR/3 laryngoscop*):ti,ab,kw | TS=(direct NEAR/3 laryngoscop* OR DL OR conventional NEAR/3 laryngoscop* OR Macintosh NEAR/3 laryngoscop* OR Miller NEAR/3 laryngoscop*) |
