# Supplementary material for: Different classes of videoscopes and direct laryngoscopes for double-lumen tube intubation in thoracic surgery: A systematic review and network meta-analysis
Source: PLoS One. 2020 Aug 28;15(8):e0238060. doi: 10.1371/journal.pone.0238060 (PMC7455027; doi:10.1371/journal.pone.0238060)
Supplement: S2 Table — (DOCX) [file pone.0238060.s003.docx]

S2 Table. Clinical characteristics for enrolled studies.

| Study | Year | Group | Age | Sex (male/female) | BMI(kg/m^2^) | MPC score (Ⅰ/Ⅱ/Ⅲ/Ⅳ) |
| --- | --- | --- | --- | --- | --- | --- |
| Ajimi et al. | 2018 | Airtraq n = 30 | 59 ± 3 | 23/7 | 22 ± 1 | 11/19/0/0 |
|  |  | AWS-200 n = 30 | 61 ± 3 | 20/10 | 21 ± 1 | 22/8/0/0 |
| Bakshi et al. | 2019 | McGrath MAC n = 37 | 46.9 ± 17 | 25/12 | 21.8 ± 3 | 29/8/0/0/ |
|  |  | Macintosh n = 37 | 49.8 ± 16 | 23/14 | 23 ± 3 | 23/14/0/0 |
| Belze et al. | 2017 | Glidescope n = 36 | 68 ±9.9 | 28/8 | 26.6 ± 5.1 | 1/10/19/6 |
|  |  | Airtraq n = 36 | 68.1 ± 9.7 | 29/7 | 27.4 ± 4.7 | 2/7/24/3 |
| Bensghir et al. | 2010 | Glidescope n = 34 | 41.8 ± 9 | 28/6 | 24 ± 2.9 | 28/8/0/0 |
|  |  | Macintosh n = 34 | 44.6 ± 10 | 29/5 | 22.98 ± 2.19 | 24/10/0/0 |
| H. Kido et al. | 2015 | McGrath MAC n = 25 | 67.9 ± 15 | 16/9 | 21.9 ± 4.6 | 5/18/2/0 |
|  |  | Macintosh n = 25 | 66.6 ±11.3 | 15/10 | 22.3 ± 3.2 | 9/16/0/0 |
| Hamp et al. | 2015 | Airtraq n = 17 | 63.4 ± 9.3 | 11/9 | not mentioned | 7/10/2/1 |
|  |  | Macintosh n = 20 | 56.8 ± 10.6 | 9/8 | not mentioned | 4/9/4/0 |
| Hsu et al. | 2012 | Glidescope n = 30 | 40.1 ± 18.7 | 7/23 | 21.3 ± 3.4 | 1/27/2 |
|  |  | Macintosh n = 30 | 37.2 ± 15.4 | 11/19 | 23 ± 5.6 | 3/27/0/0 |
| Hsu et al. | 2013 | Trachway n = 30 | 40 ± 15 | 20/10 | 21 ± 4 | 3/20/7/0 |
|  |  | Macintosh n = 30 | 47 ± 15 | 22/8 | 23 ± 4 | 5/21/4/0 |
| Huang et al. | 2020 | Glidescope n = 29 | 58 ± 8.8 | 11/18 | 23.3 ± 3.3 | 17/11/0/0 |
|  |  | C-MAC n = 30 | 57.2 ± 9.6 | 18/12 | 22.8 ± 2.7 | 17/13/0/0 |
|  |  | Macintosh n = 30 | 54.6 ± 11.8 | 20/10 | 24.3 ± 3.8 | 19/11/0/0 |
| Jiang et al. | 2011 | Airtraq n = 29 | 57.3 ± 10.9 | 22/7 | not mentioned | 3/18/8/0 |
|  |  | Macintosh n = 29 | 57 ± 11.4 | 20/9 | not mentioned | 7/16/6/0 |
| Lin et al. | 2012 | CEL-100 n = 83 | 58.2 ± 9.6 | 55/28 | 22.9/2.7 | 40/36/7/0 |
|  |  | Macintosh n = 82 | 57.6 ± 9.4 | 52/30 | 23.1/2.8 | 45/31/6/0 |
| M.R. El-tahan et al. | 2018 | Macintosh n = 32 | 27.5 ± 9.8 | 19/13 | not mentioned | 17/13/2/0 |
|  |  | Glidescope n = 34 | 39.9 ± 17.5 | 26/8 | not mentioned | 12/18/4/0 |
|  |  | Airtraq n = 35 | 33.8 ± 13.4 | 31/4 | not mentioned | 11/16/8/0 |
|  |  | King Vision n = 32 | 31.3 ± 14.8 | 27/5 | not mentioned | 14/12/6/0 |
| Risse et al. | 2020 | Glidescope n = 34 | 66 (58-75) | 25/9 | 25.2(24.1-29.1 | 14/14/6/0 |
|  |  | Macintosh n = 31 | 60 (52-65) | 25/6 | 25.7(24.2-30.8) | 11/16/4/0 |
| Russell et al. | 2013 | Glidescope n = 35 | 59 ± 12 | 15/20 | 26 ± 5 | 15/13/7/0 |
|  |  | Macintosh n = 35 | 62 ± 14 | 18/17 | 26 ± 4 | 22/11/2/0 |
| Shah et al. | 2016 | Storz C-Mac D-blade  n = 29 | 54.6 ± 11.1 | 22/8 | not mentioned | not mentioned |
|  |  | Macintosh n = 30 | 52.1 ± 12.7 | 20/10 | not mentioned | not mentioned |
| Wan et al. | 2016 | McGrath n = 45 | 52 ± 11 | 35/9 | 23 ± 3 | 25/16/3 |
|  |  | Airtraq n = 45 | 48 ± 14 | 32/11 | 22 ± 3 | 29/12/2/0 |
| Wasem et al. | 2013 | Airtraq n = 30 | 63 ± 10 | 22/8 | 27.4 ± 2.8 | 11/18/1/0 |
|  |  | Macintosh n = 30 | 55 ± 19 | 19/11 | 27.1 ± 6.2 | 12/15/3/0 |
| Xu et al. | 2015 | Shikani n = 30 | 50.1 ± 11.1 | 15/16 | 23.4 ± 3 | 6/20/4/0 |
|  |  | Macintosh n = 30 | 46.3 ± 16.1 | 17/13 | 24 ± 4.9 | 8/17/5/0 |
| Yang et al. | 2013 | OptiScope n = 198 | 62(20-78) | 140/58 | 23.2 ± 2.9 | 69/109/19/1 |
|  |  | Macintosh n = 199 | 61(25-79) | 150/49 | 23.4 ± 3.1 | 67/111/18/2 |
| Yao et al. | 2015 | McGrathseries5 n = 48 | 47.6 ± 13.8 | 33/15 | 22 ± 3.4 | 27/18/3/0 |
|  |  | Macintosh n = 48 | 47.8 ± 16.3 | 33/15 | 21.9 ± 3 | 28/18/2/0 |
| Yi et al. | 2013 | Macintosh n = 35 | 55 ± 18 | 20/15 | 24 ± 4 | 18/15/2/0 |
|  |  | Glidescope n = 35 | 56 ± 13 | 22/13 | 24 ± 4 | 11/20/4/0 |
| Yi et al. | 2015 | Airtraq n = 36 | 56 ± 15 | 22/13 | not mentioned | 17/13/5/0 |
|  |  | Glidescope n = 35 | 55 ± 13 | 21/14 | not mentioned | 18/14/3/0 |
| Yoo et al. | 2018 | Macintosh n = 22 | 47(43-53) | 14/8 | not mentioned | 12/7/3/0 |
|  |  | McGrath n = 22 | 48(46-55) | 14/8 | not mentioned | 13/4/4/1 |
