# Supplementary material for: Different classes of videoscopes and direct laryngoscopes for double-lumen tube intubation in thoracic surgery: A systematic review and network meta-analysis
Source: PLoS One. 2020 Aug 28;15(8):e0238060. doi: 10.1371/journal.pone.0238060 (PMC7455027; doi:10.1371/journal.pone.0238060)
Supplement: Supplementary file 5 [file pone.0238060.s005.docx]

	PubMed	No.	Embase	No.	Cochrane Trials (SR 0)	No.	WoS	No.	Kmbase	No.	Scopus	No.
#1	(Intubation, Intratracheal[mh] OR Intubat*[tw]) AND (double lumen[tw] OR dual lumen[tw])	1,208	('endotracheal intubation'/exp OR 'Nasotracheal Intubation'/exp OR intubat*:ti,ab) AND ('double lumen tube'/exp OR (double NEAR/3 lumen OR dual NEAR/3 lumen):ti,ab)	1,339	([mh "Intubation, Intratracheal"] OR Intubat*:ti,ab,kw) AND (double NEAR/3 lumen OR dual NEAR/3 lumen):ti,ab,kw	334	TS=(Intubat* AND (double NEAR/3 lumen OR dual NEAR/3 lumen))	571	intubat*		TITLE-ABS-KEY(Intubat* AND (double W/3 lumen OR dual W/3 lumen))	1,496
#2	(Laryngoscopy[mh] OR Laryngoscopes[mh]) AND Video Recording[mh]	1,515	videolaryngoscopy/exp OR (laryngoscopy/exp AND videorecording/exp)	1,349	([mh Laryngoscopy] OR [mh Laryngoscopes]) AND [mh "Video Recording"]	208						
#3	videolaryngoscop*[tw] OR video-laryngoscop*[tw] OR VL[tw] OR VLS[tw] OR (video[tw] AND laryngoscop*[tw]) OR Airtraq[tw] OR Bullard[tw] OR Pentax[tw] OR Glidescope[tw] OR McGrath[tw] OR Storz[tw] OR Venner[tw] OR King Vision[tw] OR KingVision[tw] OR VividTrac[tw] OR CoPilot[tw] OR Ue scope[tw] OR stylet[tw] OR TruView[tw] OR Airway Scope[tw] OR AWS[tw] OR CEL[tw] OR C-MAC[tw] OR A.P. Advance[tw] OR EndoSTROB[tw] OR Glidescope[tw] OR VLP-100[tw] OR LMA CTrach[tw]	21,660	(videolaryngoscop* OR video-laryngoscop* OR VL OR VLS OR ((video OR indirect) NEAR/3 laryngoscop*) OR Airtraq OR Bullard OR Pentax OR Glidescope OR McGrath OR Storz OR Venner OR 'King Vision' OR KingVision OR VividTrac OR CoPilot OR 'Ue scope' OR stylet OR TruView OR 'Airway Scope' OR AWS OR CEL OR C-MAC OR 'A.P. Advance' OR EndoSTROB OR Glidescope OR VLP-100 OR 'LMA CTrach'):ti,ab	29,944	(videolaryngoscop* OR video-laryngoscop* OR VL OR VLS OR ((video OR indirect) NEAR/3 laryngoscop*) OR Airtraq OR Bullard OR Pentax OR Glidescope OR McGrath OR Storz OR Venner OR "King Vision" OR KingVision OR VividTrac OR CoPilot OR "Ue scope" OR stylet OR TruView OR "Airway Scope" OR AWS OR CEL OR C-MAC OR "A.P. Advance" OR EndoSTROB OR Glidescope OR VLP-100 OR "LMA CTrach"):ti,ab,kw	3,796	TS=(videolaryngoscop* OR video-laryngoscop* OR VL OR VLS OR ((video OR indirect) NEAR/3 laryngoscop*) OR Airtraq OR Bullard OR Pentax OR Glidescope OR McGrath OR Storz OR Venner OR "King Vision" OR KingVision OR VividTrac OR CoPilot OR "Ue scope" OR stylet OR TruView OR "Airway Scope" OR AWS OR CEL OR C-MAC OR "A.P. Advance" OR EndoSTROB OR Glidescope OR VLP-100 OR "LMA CTrach")	27,846	(laryngoscop* AND video) OR video-laryngoscop* OR videolaryngoscop*		TITLE-ABS-KEY(videolaryngoscop* OR video-laryngoscop* OR VL OR VLS OR ((video OR indirect) W/3 laryngoscop*) OR Airtraq OR Bullard OR Pentax OR Glidescope OR McGrath OR Storz OR Venner OR "King Vision" OR KingVision OR VividTrac OR CoPilot OR "Ue scope" OR stylet OR TruView OR "Airway Scope" OR AWS OR CEL OR C-MAC OR "A.P. Advance" OR EndoSTROB OR Glidescope OR VLP-100 OR "LMA CTrach")	40,492
#4	direct laryngoscop*[tw] OR DL[tw] OR conventional laryngoscop*[tw] OR (Macintosh[tw] AND laryngoscop*[tw]) OR (Miller[tw] AND laryngoscop*[tw])	129,458	(direct NEAR/3 laryngoscop* OR DL OR conventional NEAR/3 laryngoscop* OR Macintosh NEAR/3 laryngoscop* OR Miller NEAR/3 laryngoscop*):ti,ab OR 'device comparison'/exp	242,319	(direct NEAR/3 laryngoscop* OR DL OR conventional NEAR/3 laryngoscop* OR Macintosh NEAR/3 laryngoscop* OR Miller NEAR/3 laryngoscop*):ti,ab,kw	18,785	TS=(direct NEAR/3 laryngoscop* OR DL OR conventional NEAR/3 laryngoscop* OR Macintosh NEAR/3 laryngoscop* OR Miller NEAR/3 laryngoscop*)	128,334			TITLE-ABS-KEY(direct W/3 laryngoscop* OR DL OR conventional W/3 laryngoscop* OR Macintosh W/3 laryngoscop* OR Miller W/3 laryngoscop*)	3,224
#5	#2 OR #3 OR #4	149,786	#2 OR #3 OR #4	270,355	#2 OR #3 OR #4	21,555	#3 OR #4	154,476			#3 OR #4	42,694
#6	#1 AND #5	123	#1 AND #5	168	#1 AND #5	94	#1 AND #5	97	#1 AND #3	54	#1 AND #5	138
amount		123		168		94		97		54		138
amount after delete duplication		123		60		38		15		50		26
